# Editorial: Current advancements in real-time plant pathogen diagnostics: from lab assays to in-field detection

**DOI:** 10.3389/fpls.2023.1255654

**Published:** 2023-08-04

**Authors:** Ravinder Kumar, Milan Kumar Lal, Pramod Prasad, Rahul Kumar Tiwari

**Affiliations:** ^1^ Division of Plant Protection, ICAR-Central Potato Research Institute, Shimla, Himachal Pradesh, India; ^2^ Plant Pathology, ICAR-Indian Institute of Wheat and Barley Research, Regional Station, Shimla, Himachal Pradesh, India

**Keywords:** plant disease, plant pathogen, detection, field level diagnostics, fungi, bacteria, viruses

## Introduction

Food security and increased global population have driven agricultural activities to strive for higher productivity. There are however numerous invasive plant pathogens that cause plant diseases and reduce crop yields, including viruses, fungi, nematodes, mycoplasma, bacteria and others (Raigond et al., 2022; Chikh-Ali and Karasev, 2023; Thakur et al., 2023). Approximately, $220 billion is lost to the global economy each year as a result of plant diseases caused by these pathogens (Lal et al., 2021; Tiwari et al., 2021; Raigond et al., 2022). Diseases of plants are historically diagnosed by looking at their symptoms and appearance, often at advanced stages when they are difficult to manage and treat. Single or mixed infections can occur with pathogens (Shah et al., 2020). The most effective way to manage plant diseases is to use healthy, disease-free plants. To ensure food security and minimize crop losses, continuous and immediate monitoring and pathogen identification efforts must be undertaken before planting crops in the field (Kumar et al., 2017; Bhardwaj et al., 2019; Kumar et al., 2020b; Tiwari et al., 2020; Tiwari et al., 2022; Rahman et al., 2023).

Increasing sensitivity and specificity of disease monitoring in the field have been enabled by advances in real-time diagnostics. There are several techniques available for detecting plant pathogens, such as enzyme-linked immunosorbent assay (ELISA), polymerase chain reaction (PCR), real-time PCR, fluorescence *in situ* hybridization (FISH), and flow cytometry. However, these methods often suffer from certain limitations, including being time-consuming, costly, and requiring highly skilled personnel for their execution (Kumar et al., 2020a; Kumar et al., 2022). Therefore, plant pathology is focusing on rapid, accurate, and cost-effective diagnostics, especially for emerging diseases or elusive pathogens with subtle initial symptoms.

Moreover, diagnostic laboratories have become increasingly dependent on innovative diagnostic tools designed for field use in an interconnected global environment. The tools used ensure that instruments and techniques are operationally relevant. Nanotechnology and biosensor-based diagnostics, along with portable systems integrated with the Internet of Things (IoT), have revolutionized the field of pathogen detection. These advancements have led to the development of isothermal amplification-based nucleic acid visual detection systems, which are highly efficient in identifying pathogens. Key technologies within this framework include Loop-mediated isothermal amplification (LAMP), rolling circle amplification (RCA), recombinase polymerase assay (RPA), and CRISPR/Cas (Raigond et al., 2020; Kumar et al., 2021; Watpade et al., 2023).

## The latest advancements in real-time plant pathogen diagnostics

To prevent an outbreak of plant diseases, farmers worldwide must detect them quickly and accurately. Rapid diagnosis of pathogens is, therefore, necessary to reduce yield losses. We gathered the latest research on field-level diagnostics for real-time identification and timely management of plant diseases in crops for this Research Topic. The eleven research articles on diagnostics cover a broad range of topics, including the diagnosis and management of bacterial, fungal, viral, phytoplasma, and nonparasitic diseases.

Cheng et al. identified the proteins unaffected by Pst DC3000 infection by mass spectrometry-based label-free quantification (LFQ) and demonstrated the capability of this to quantify protein abundance and the possibility of extending protein expression studies to transcripts in Arabidopsis. Ren et al. tested 100 inter simple sequence repeats (ISSR) primers and generated a species-specific fragment (515 bp) with ISSR 827 against *Tilletia caries*. In addition, they developed a super-sensitive quantitative real-time polymerase chain reaction (qRT-PCR) with a detection limit of 2.4 fg/μL, and droplet digital PCR (ddPCR) with a detection limit of 0.24 fg/μL. In a study by Logeshwari et al., a LAMP was developed for highly sensitive detection of *Sarocladium oryzae* at concentrations as low as 10 fg in 30 minutes at 65°C. LAMP was validated using live infected tissues, weeds and seeds collected from different locations in Tamil Nadu. Using reverse transcription recombinase-amplification (RT-RAA) and CRISPR/Cas12a-based lateral flow assays, Lei et al. developed a rapid detection method that can detect 2.5 copies of the coat protein gene of MCMV, using 0.96 pg of total RNA extracted from maize leaves infected with MCMV. To make the method more feasible for field detection, crude virus extraction of plant RNA combined with one-tube RT-RAA/CRISPR-Cas12a reaction was implemented on a portable metal incubator (37-42 ^0^C).

Awan et al. conducted antifungal bioassays, and the metabolites extracted from BS-01 exhibited the most potent inhibition of fungal biomass. The extracellular metabolites displayed an impressive inhibition range of 69-98%, while the intracellular metabolites showed inhibition ranging from 48% to 85%. In comparison, the metabolites extracted using n-hexane demonstrated inhibition percentages of 63-88% for extracellular metabolites and 35-62% for intracellular metabolites. Similarly, the use of dichloromethane resulted in inhibition percentages of 41-74% for extracellular metabolites and 42-74% for intracellular metabolites. In growth chamber bioassays, both foliar application and seed application of BS-01 significantly reduced *Alternaria solani* load on inoculated tomato foliage. To improve Plant parasitic nematodes (PPNs) identification and detection, Shao et al. reviewed the latest research advances and diagnostic approaches and techniques. Morphological characters alone are not sufficient to identify PPNs because they often have interspecific overlays and wide intraspecific variations. PPNs can now be diagnosed directly in the field using newly developed isothermal amplification technologies and remote sensing methods. Lal et al. studied the worldwide research on real-time PCR-based pathogen detection from 2001 to 2021 that was used for any diagnostic assay or gene expression level study. According to the analysis, research on RT-PCR-based pathogen detection is booming and should be strengthened by using modern diagnostic tools and collaboration among labs equipped with the necessary equipment. Using crude sap lysed in 0.5M NaOH solution as a template and purified DNA/cDNA as a primer, Kishan et al. developed an isothermal-based recombinase polymerase amplification (RPA) method for the detection of Grapevine geminivirus A (GGVA) in grapevine samples. This assay has the advantage of not requiring purification or isolation of viral DNA and can be performed at a wide range of temperatures (18-46°C) for 10-40 minutes, making it an effective and rapid way to detect grapevine GGVA. Buttar et al. demonstrated that three applications of Trifloxystrobin+ Tebuconazole 75% WG @ 0.07% were the most effective against pod rot disease on two mungbean cultivars, ML 2056 and SML 668. ML 2524, among the tested genotypes, exhibited resistance to pod rot disease, with an incidence of 15.62% and a severity of 7.69%. A new protocol was developed by Moran et al. that does not require nucleic acid purification or specialized equipment, making it ideal for field use. Primer and probe targeting a region of the fusA gene show 94-100% specificity both *in vitro* and in silico for the `Ca. Liberibacter´ species associated with HLB. HLB-infected plant and insect material can be detected with a reliable limit of 101 copies per microliter using the new protocol. Chauhan et al. studied biochemical mechanisms associated with cotton leaf curl disease (CLCuD) resistance. High-diseased plants of the susceptible hybrid HS 6 had a value of 0.7 mg g-1 at 60 DAS. At 90 DAS, resistant cultivars exhibited the highest phenol content (0.70 mg g-1). HS 6 (9.4 mg g-1) and RCH 134 BG-II (10.5 mg g-1) showed the lowest protein activity at 120 DAS. CLCuV protection in cotton begins with protein activity, one of the primary biochemical compounds. In cotton, phenol and tannin are the secondary levels of defense, showing significant increases in their levels while imparting resistance against CLCuV.

## Conclusions and perspectives

In conclusion, the Research Topic addresses the critical need for early detection and accurate diagnosis of plant pathogens to mitigate crop losses and ensure food security. It emphasizes the development of diagnostic techniques and tools that are simple, specific, rapid, and cost-effective. The focus was on advancing field-deployable molecular diagnostics that enable on-the-spot pathogen detection and immediate response. Isothermal amplification techniques such as RPA have gained prominence in field-deployable diagnostics. This technique amplifies targeted nucleic acids of pathogens at a constant temperature, eliminating the need for complex thermal cycling equipment. They are simple, fast, and robust, making them suitable for on-site detection even in resource-limited settings. CRISPR/Cas technologies have also shown promise in plant pathogen detection. These methods leverage the Cas enzyme’s ability to target and cleave specific sequences in the pathogen’s DNA or RNA. Coupled with a detection system, CRISPR-based diagnostics enable rapid identification of pathogens in the field, facilitating real-time disease monitoring and control. Overall, these advancements in field-deployable molecular diagnostics, including portable systems interconnected with isothermal amplification techniques like LAMP and RPA, and CRISPR/Cas technologies, are revolutionizing the field of plant pathology. They provide rapid, reliable, and on-the-spot pathogen detection capabilities, empowering farmers, researchers, and agricultural professionals to make informed decisions and take immediate action to protect crops from the devastating effects of plant pathogens.

## Author contributions

RK: Conceptualization, Resources, Visualization, Writing –original draft, Writing – review & editing, MKL: Conceptualization, Resources, Visualization, Writing – original draft, Writing – review & editing, PP: Conceptualization, Resources, Visualization, Writing – original draft, Writing – review & editing. RKT: Data curation, Formal Analysis, Investigation, Methodology, Resources, Software, Writing – original draft, Writing – review & editing.
